# Case report: Sclerosing encapsulating peritonitis in a cat with disseminated pancreatic adenocarcinoma of presumed ductal origin

**DOI:** 10.3389/fvets.2024.1406223

**Published:** 2024-07-31

**Authors:** Chaerin Kim, Sanggu Kim, Jinhyeong Park, Dohee Lee, Yeon Chae, Taesik Yun, Dongwoo Chang, Byeong-Teck Kang, Sungin Lee, Soochong Kim, Hakhyun Kim

**Affiliations:** ^1^Laboratory of Veterinary Internal Medicine, College of Veterinary Medicine, Chungbuk National University, Cheongju, Republic of Korea; ^2^Laboratory of Veterinary Pathology, College of Veterinary Medicine, Chungbuk National University, Cheongju, Republic of Korea; ^3^Laboratory of Veterinary Imaging, Veterinary Teaching Hospital, College of Veterinary Medicine, Chungbuk National University, Cheongju, Republic of Korea; ^4^Laboratory of Veterinary Surgery, College of Veterinary Medicine, Chungbuk National University, Cheongju, Republic of Korea

**Keywords:** peritonitis, pancreatic tumor, adipose tissue, feline, metastasis

## Abstract

A 9-year-old, neutered male, domestic short-haired cat was referred for recurrent ascites of unknown etiology over a week. Physical examination revealed abdominal distension and ultrasonography revealed a large volume of ascites throughout the abdominal cavity; this was interpreted as modified transudate. The mesentery and abdominal fat were hyperechoic and edematous. Fat tissue was assessed using fine-needle aspiration cytology, and adipocytes, fat-phagocytizing macrophages, and neutrophils were identified. Computed tomography revealed a pancreatic mass connected to the left pancreatic leg. Exploratory laparoscopy confirmed nodular masses and organ adhesions, leading to a tentative diagnosis of sclerosing encapsulating peritonitis. The cat was administered prednisolone, vitamin E, and tamoxifen but died 22 days after the initial therapy. Necropsy revealed a multi-lobulated pancreatic tumor (10 × 10 cm) tightly attached to the stomach and intestine, with a large amount of ascites. The peritoneum, stomach, intestine, and mesentery were covered with numerous disseminated nodules of various sizes (1–5 mm diameter). Microscopically, the tumor consisted of extensive adipose tissue, locally extensive inflammatory infiltrates, fibrous connective tissue, and small invasive proliferative glands. Well-defined small irregular glands composed of single-layered epithelial cells that appear to be of ductal origin were surrounded by an abundant desmoplastic stroma. Neoplastic nodules were widespread in the liver, stomach, peritoneum, mesentery, mesenteric lymph nodes, lungs, and urinary bladder. Immunohistochemistry revealed that the neoplastic glands were positive for pan-cytokeratin, confirming the pancreatic epithelial origin of the tumor. This is the first report of sclerosing encapsulating peritonitis accompanied by aggressive pancreatic adenocarcinoma of presumed ductal origin and extensive metastasis in a cat.

## 1 Introduction

Sclerosing encapsulating peritonitis (SEP) is a chronic form of peritonitis characterized by adhesions of the abdominal organs, fibrosis, and thickening of the visceral and parietal peritoneum (1). The clinical signs result from intestinal adhesions and peritoneal irritation, which present with non-specific clinical manifestations. Most cases involve chronic peritoneal effusions that may be accompanied by digestive symptoms such as vomiting, diarrhea, anorexia, weight loss, or abdominal pain (2). In humans, the major underlying causes are peritoneal dialysis, abdominal surgery, drug administration, and abdominal tumors (3). However, it is uncommon in dogs and cats, and the etiology of SEP is not fully understood but is thought to be multifactorial (4–6). Some animals diagnosed with SEP have a history of abdominal foreign bodies or chronic bacterial infections; however, the cause of SEP is idiopathic (7). Recent studies have reported various tumor types as potential contributors to its onset (8, 9). However, there have been no reports of concurrent SEP and pancreatic ductal adenocarcinoma (PDAC), an aggressive tumor with a high metastasis rate and poor prognosis, particularly in cats with abdominal effusion (10). This case report describes a presumed PDAC with concurrent SEP in a cat with recurrent massive abdominal effusion.

## 2 Case presentation

A 9-year-old, castrated male, domestic short-haired cat weighing 5.94 kg presented with hyporexia for a week. Ascites was detected during the initial examination. The cat had a history of a diet change five days prior to presentation, which was accompanied by symptoms including soft stools and vomiting. Approximately 500 mL of peritoneal fluid was removed via abdominocentesis on the day of presentation, after which there was no further vomiting and an improvement in the fecal consistency was noted. However, a decline in vitality and appetite occurred three days later, prompting second abdominocentesis to remove 420 mL of peritoneal fluid.

Blood analysis, radiography, ultrasonography, fluid analysis, and ascitic fluid bacterial culture were performed at a referring hospital. Blood analysis revealed no remarkable findings except for an elevated serum albumin-to-globulin ratio (0.8). The serum feline pancreatic lipase level was mildly elevated (5.7 ng/mL; reference interval [RI] = < 3.5 ng/mL). Abdominal ultrasonography revealed edematous changes in the omentum and multiple hypoechoic nodules, each measuring <1 cm, in the peritoneum. Because both aerobic and anaerobic bacterial cultures of peritoneal fluid yielded negative results, the likelihood of the ascites being attributed to infection was excluded. Additionally, the serum concentration of N-terminal pro-brain natriuretic peptide was within the normal range, excluding heart disease as a potential cause of ascites. Despite thorough examination, the cause of the clinical signs and recurrent ascites was not found.

**Figure 1:**
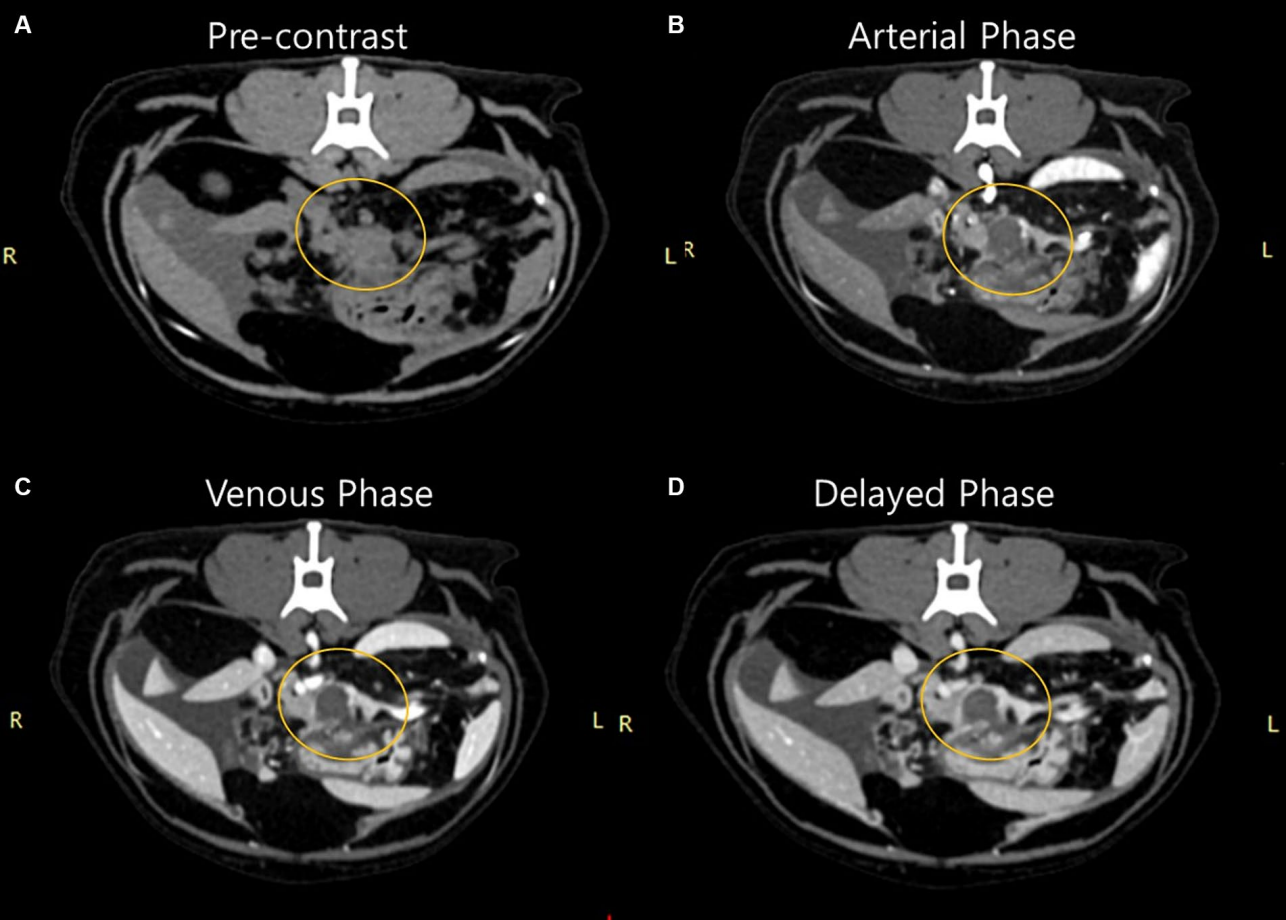
Findings of the first computed tomography scan of a 9-year-old cat presenting with recurrent ascites of unknown etiology. Triphasic images before **(A)** and after **(B)** imaging in the ventrodorsal position. **(A)** Pre-contrast stage, **(B)** arterial phase, **(C)** venous phase, **(D)** delayed phase. A mass showing connectivity with the left leg of the pancreas was observed; it was ill-defined, amorphous with an irregular margin, and an ambiguous ventral margin. No significant internal contrast enhancement was observed in any phase after imaging, and it was homogenously hypodense compared with normal pancreatic parenchyma.

**Figure 2:**
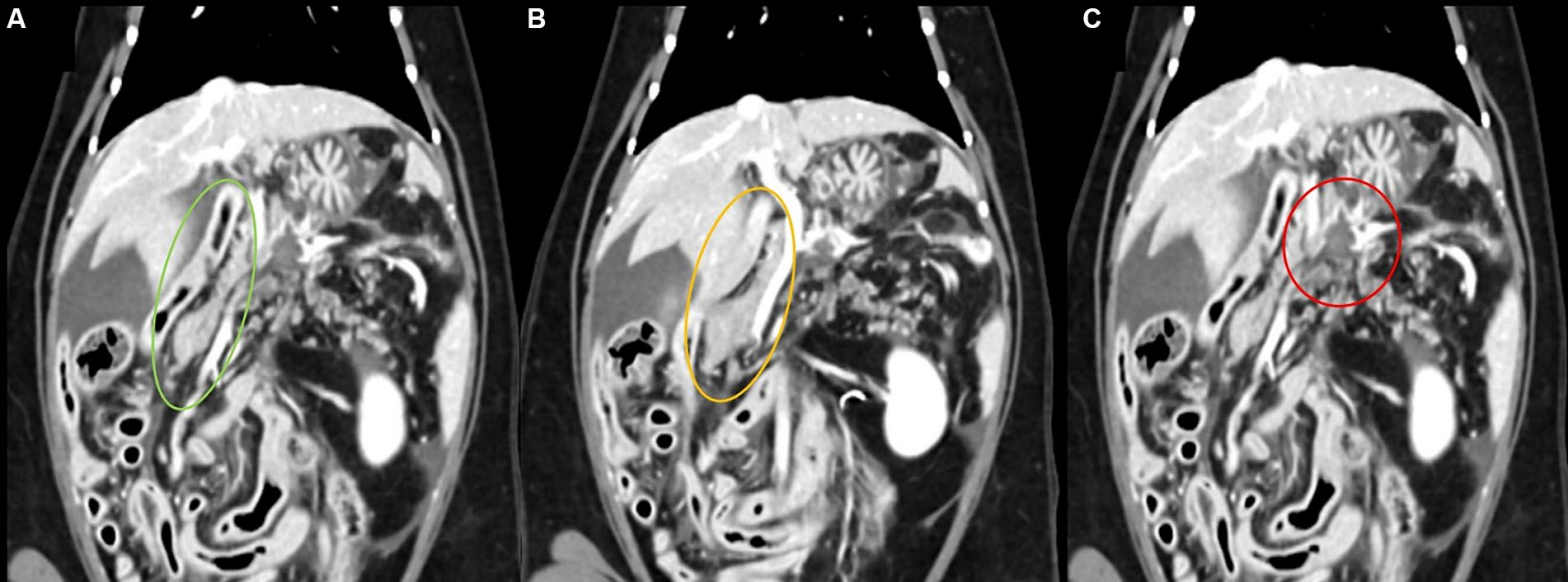
Findings of the first computed tomography scan of a 9-year-old cat presenting with recurrent ascites of unknown etiology. Triphasic images from delay phase in the dorsal plane **(A–C)**. **(A)** The right leg of the pancreas was well observed with relatively normal shape and strong contrast enhancement (green circle). **(B)** After imaging, the periphery of the mass received blood flow from the splenic artery, which branches from the celiac artery, and rim enhancement (orange circle) was observed in the portal vein and lag phase. **(C)** Intra-abdominal lymph nodes showed homogenous contrast enhancement (red circle) with mild hypertrophy while maintaining their normal shape.

The first computed tomography (CT) scan was performed at the referring hospital to determine the cause of ascites. A mass demonstrating connectivity with the left leg of the pancreas was identified (Figure 1). The pattern was observed to be ill-defined and amorphous with irregular margins. It was approximately 1.18 × 1.16 × 1.12 cm (width × height × length) in size, and the ventral margin of the lesion was vague. No significant internal contrast enhancement was noted in any phase after imaging, and the lesion appeared homogenously hypodense compared with the normal pancreatic parenchyma. Lymph nodes, including the splenic, hepatic, pancreaticoduodenal, and jejunal lymph nodes, receiving lymphatic drainage from the mass lesion and pancreas, were mildly enlarged. Severe fat-stranding was observed around the mass, involving the surrounding peritoneum with nodules in a diffusely aggregated pattern, presumably due to fat necrosis (Figure 2). Additionally, a substantial amount of free fluid was noted in the abdominal cavity, but not in the pleural cavity, where the retro-abdominal cavity appeared intact with normal fat density. The abdominal wall exhibited irregularities with mixed nodular patterns, and peritoneal and mesenteric thickening was observed.

The cat was referred 4 days after CT. Physical examination revealed mild dehydration (5–6%). In complete blood cell counts (ProCyte Dx, IDEXX Laboratories, Westbrook, ME, USA), low lymphocyte (0.74 × 10^3^/μL, RI = 0.92–6.88 × 10^3^/μL) and high reticulocyte (82.4 × 10^3^/μL, RI = 3.0–50.0 × 10^3^/μL) counts were detected. The serum biochemistry profile (Hitachi XYZ, Hitachi Ltd., Tokyo, Japan) showed elevated serum creatinine (2.0 × 10 mg/dL, RI = 0.7–1.8 × 10 mg/dL), glucose (240 mg/dL, RI = 75–199 mg/dL), and lactate (5.28 mmol/L, RI = 0.50–2.50 mmol/L). In addition, serum symmetric dimethylarginine (15 µg/dL, RI = 0–14 µg/dL; IDEXX Laboratories, Westbrook, ME, USA) and amyloid A concentration (52.86 mg/L, RI = 0–10 mg/L; Bionote, Gyeoggi-do, South Korea) were increased. In the serum electrolyte profile (Roche Cobas c 9,180, Roche Diagnostics, Basel, Switzerland), a low sodium concentration (143 mmol/L, RI = 145–158 mmol/L) and a normal total calcium concentration (9.0 mg/dL, RI = 8.2–10.8 mg/dL) were detected. Abdominal ultrasonography revealed a large amount of ascites between the liver lobes, around the kidneys, and in the anterior bladder. The mesentery and fat tissue were hyperechoic and edematous (Supplementary Figure S1). The left leg of the pancreas was not clearly visualized; ascites was observed around the left end and the pancreatico-duodenal lymph nodes were enlarged. Approximately 320 mL of peritoneal fluid was drained via ultrasound-guided abdominocentesis. The peritoneal fluid appeared reddish and cloudy upon macroscopic examination (Supplementary Figure S2A). Cytological examination of the peritoneal fluid revealed modified transudate, predominantly composed of neutrophils and macrophages, with visible lymphoid cells (total nucleated cell count, 2,680 cells/μL; total protein: 3.4 mg/dL) (Supplementary Figure S2B). The albumin-to-globulin ratio of the peritoneal fluid was 0.9, and the Rivalta test result was negative, suggesting that feline infectious peritonitis was unlikely. Abdominal fat fine-needle aspiration (FNA) was performed under ultrasonographic guidance. Adipocytes and fat-phagocytizing macrophages were identified, and inflammation and macrophage infiltration were observed in the fat tissue. The specific feline pancreatic lipase value (IDEXX Laboratories, Westbrook, ME, USA) in the serum was within the normal range (<1.00 ng/mL, RI = < 3.5 ng/mL), but in the peritoneal fluid, it was elevated beyond the normal range observed in blood (3.78 ng/mL, RI in blood = < 3.5 ng/mL). Peritoneal fluid FPL values were obtained using the same methodology as that used for serum analysis. The pancreatic lipase level ratio of abdominal fluid level to serum level was >3.78. Considering the local FNA results and elevated serum amyloid A concentration, it was suggested that the observed intra-abdominal inflammation was associated with pansteatitis. Prompt prednisolone 0.5 mg/kg q24h (Solondo®, Yuhan, Seoul, South Korea) and vitamin E 15 U/kg q24h (Grandpherol®, Yuhan, Seoul, South Korea) were administered for anti-inflammatory and antioxidant purposes, as the possibility of secondary feline pansteatitis due to vitamin E deficiency could not be ruled out (11).

Despite the anti-inflammatory treatment, there was no improvement in the clinical signs and ascites. An additional CT scan was performed on the 11th day after referral. No obvious differences were observed between it and the original CT scan. A mass showing connectivity with the left leg of the pancreas appeared on the pre-contrast images to be indistinctly delineated, as on previous imaging. However, the internal aspect of the mass after contrast administration was homogeneously hypoattenuated compared with the pancreatic parenchyma, and moderate peripheral rim enhancement was observed in the surrounding area. Severe fat stranding in the peritoneum and massive ascites were still evident. Additionally, a filling-defect-like lesion of approximately 3.48 × 2.92 × 3.52 mm (length × width × height) was observed in the blood vessel where the splenic vein flowed into the portal vein, noted in the cranio-lateral region of the mass. Possible compression of the splenic vein by the mass was considered (Supplementary Figure S3).

Based on the findings of the second CT, a pancreatic tumor or chronic peritonitis secondary to pancreatic lipase leakage was considered the cause of recurrent ascites. Exploratory laparotomy was performed to determine the cause of the ascites, and the resected mass was presumed to be a pancreatic tumor. The falciform ligament was sclerosing and thickened (Supplementary Figure S4). In addition, fibrinous adhesion of the thickened abdominal wall with adjacent organs (Supplementary Figure S4B) and a substantial amount of ascites were observed. Nodular mass lesions and adhesions were identified in the abdominal organs, including the spleen, pancreas, intestine, and peritoneum. Due to these chronic inflammatory lesions, it was impossible to distinguish between the inflammation and the tumor on macroscopic examination. Furthermore, difficulty accessing the mass prevented its accurate sampling and removal. Nodular masses in the pancreas and surrounding inflammatory fat tissue were found to be severely diffuse, with chronic interstitial pancreatitis and peripancreatic steatitis with fibrosis. The second aerobic and anaerobic bacterial cultures from the peritoneal fluid were negative. Based on these results, the cat was diagnosed with SEP.

After surgery, peripheral parenteral nutrition was initiated because of persistent anorexia in the cat. Ampibactam 22 mg/kg q12h (Sulbacin®, Donggwang, Seoul, South Korea) IV was administered to prevent secondary infection, and a Fentanyl patch 12.5 µg/kg (Durogesic D-trans Patch®, Janssen Korea, Seoul, South Korea) was used for pain management. Based on the dose of tamoxifen administered to dogs with SEP, tamoxifen 1 mg/kg q24h (Nolvadex®, AstraZeneca Korea, Seoul, South Korea) PO was added to ameliorate peritoneal fibrosis. However, on the third day of tamoxifen administration, the patient exhibited worsening hypoalbuminemia and an elevated serum amyloid A concentration. The cat developed dyspnea due to pleural effusion and subsequently died.

Necropsy findings showed that a multi-lobulated pancreatic tumor measuring 10 cm × 10 cm was tightly attached to the internal organs, including the stomach and intestines. The small and large intestines, along with the mesentery, exhibited adhesion and sclerosis, with disseminated multifocal seeding nodules of various sizes on the serosal surface (Supplementary Figure S5A). The abdominal wall was severely thickened and covered with strands of fibrinous materials and numerous disseminated nodules of various sizes (ranging from 1–5 mm in diameter). Transverse sections showed tight fibrosis and steatosis between the intestines, which compressed the intestinal lumen (Supplementary Figure S5B).

**Figure 3:**
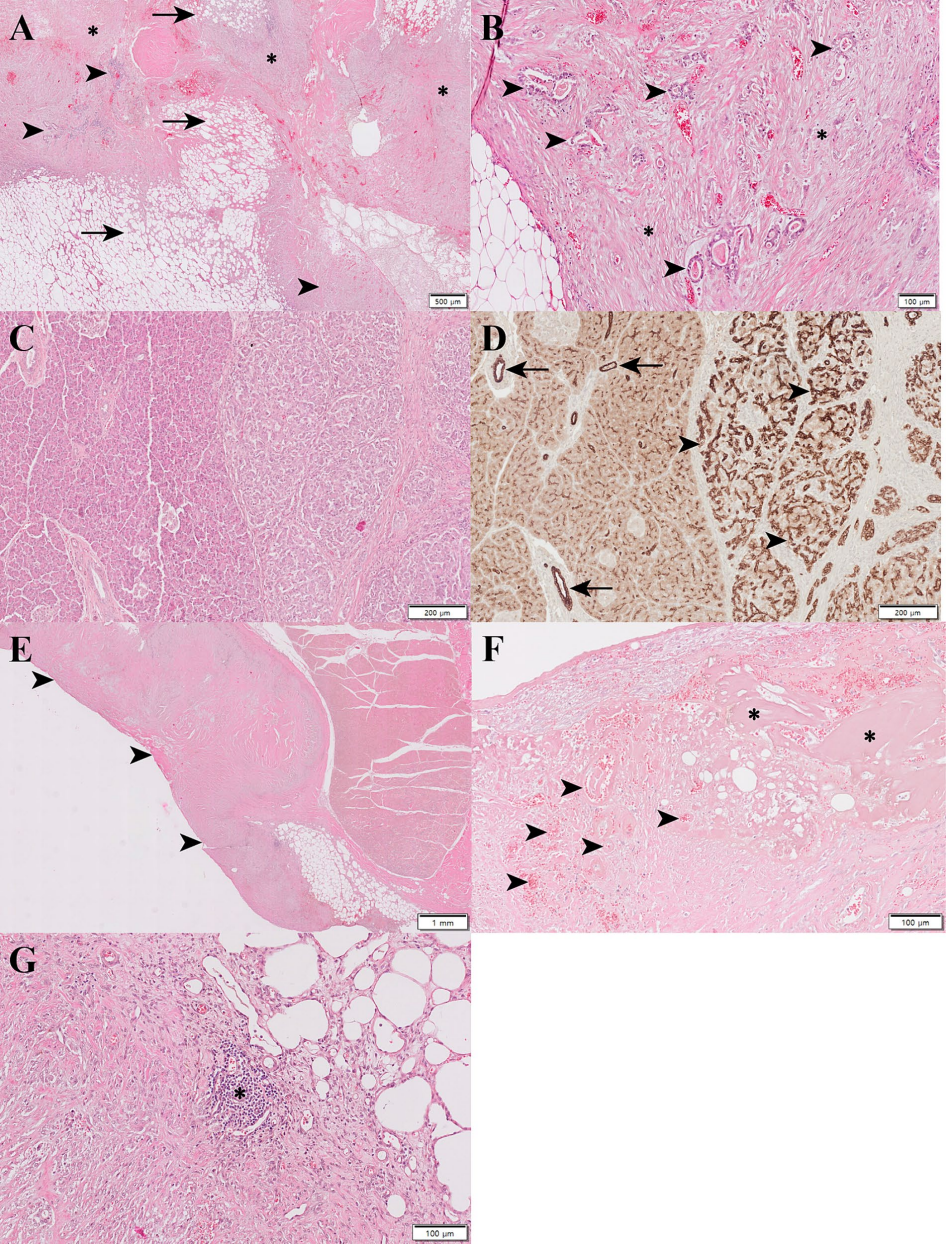
Histopathological and immunohistochemical evaluation of a pancreatic tumor in a cat. **(A)** The mass was composed of extensive adipose tissue (arrow), locally extensive inflammatory infiltrates, fibrous connective tissue (asterisk), and small invasive proliferative glands (arrowhead). **(B)** Well-defined small irregular glands composed of single-layered epithelial cells (arrowhead) were surrounded by abundant desmoplastic stroma (asterisk). **(C)** H&E staining shows normal pancreatic tissue on the left and a pancreatic tumor on the right. **(D)** In immunohistochemistry, the normal pancreatic ducts (arrows) on the left were positive for pancytokeratin. Consistently, neoplastic glands (arrowhead) on the right were positive for pancytokeratin. **(E)** Visceral peritoneum was thickened by sclerotic fibrous connective tissue (arrowhead). **(F)** Collagen, fibrin, and neovascularization were increased in peritoneum that had denuded mesothelium. **(G)** Lymphocytic inflammatory cells (asterisk) were infiltrated in sclerotic stroma.

**Figure 4:**
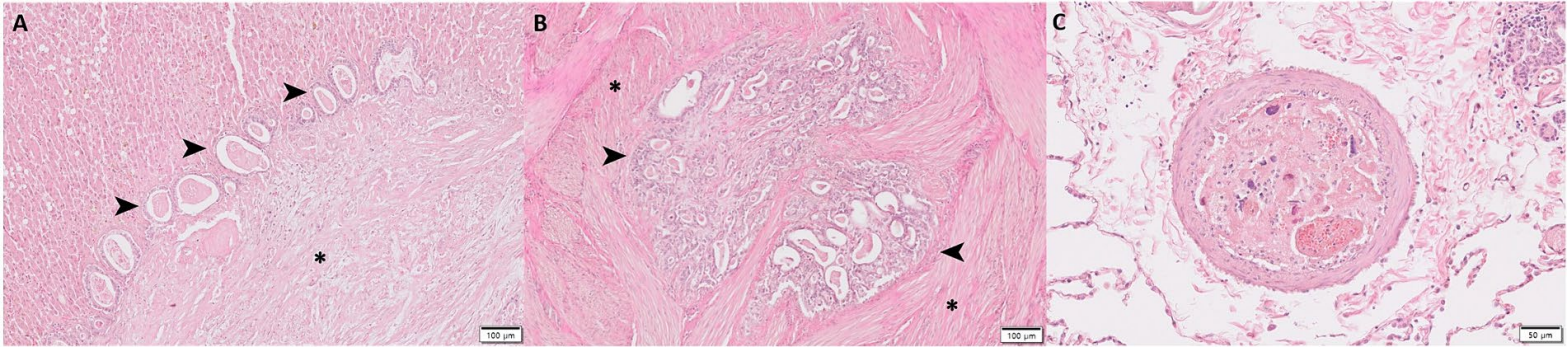
Histopathological evaluation of the metastases of pancreatic adenocarcinoma of presumed origin in the cat. Extensive implantation of neoplastic nodules (arrowhead) with desmoplasia (asterisk) was observed in the **(A)** liver and **(B)** urinary bladder. **(C)** The pulmonary blood vessel was filled with tumor emboli.

Histopathologically, the pancreatic mass was composed of extensive adipose tissue, locally extensive infiltrates of phagocytizing macrophages, fibrous connective tissue, and small invasive proliferative glands (Figure 3A). The mass showed a typical histomorphology of pancreatic ductal adenocarcinoma with well-defined, small, irregular glands composed of single-layer ductal epithelial-like cells surrounded by abundant desmoplastic stroma (Figures 3B,C). Neoplastic cells were round to oval in shape with less eosinophilic cytoplasm and no zymogen granules, and the nuclei showed chromatin clusters and prominent nucleoli. Moderate anisocytosis and anisokaryosis were present with low mitotic figures. There was no evidence of cystic formation, accumulation of mucin, and lymphatic invasion. Immunohistochemistry (IHC) was performed to further confirm the origin of the tumor cells using anti-pancytokeratin antibodies. Normal pancreatic ducts were positive for pancytokeratin, and the neoplastic glands were consistently positive for pancytokeratin (Figure 3D), further suggesting the tumor to be of the pancreatic ductal origin. In addition, lymphocytic inflammatory cells infiltration, increased collagen, fibrin, and neovascularization, and denuded mesothelium were observed in visceral peritoneum, indicating the presence of SEP (Figures 3E–G). Extensive implantation of neoplastic nodules with desmoplasia was observed in the liver, stomach, peritoneum, mesentery, mesenteric lymph nodes, and urinary bladder (Figures 4A,B). Tumor emboli were also observed in the pulmonary blood vessels (Figure 4C). Based on these results, the cat was diagnosed with SEP secondary to presumed PDAC with extensive metastasis to other organs.

## 3 Discussion

This report describes a case of presumed PDAC with concurrent SEP in a cat presenting with recurrent peritoneal effusion. SEP and PDAC are rare in cats, although SEP has been described in in a few veterinary case reports. Pancreatic adenocarcinoma is also very rare, accounting for <0.5% of all cancer cases in cats. Therefore, the ante-mortem diagnosis of PDAC and SEP is challenging owing to insufficient information in cats. We cautiously suggest that massive and recurrent ascites of unknown etiology with findings of a pancreatic mass, irregularities of the abdominal wall with mixed nodular patterns, and peritoneal and mesenteric thickening, may be a clinical clue, as shown in the present case.

The clinical manifestations of SEP are variable and non-specific. In humans, clinical symptoms occur because of intestinal obstruction, inflammation, and peritoneal adhesions. Most clinical signs are digestive and include anorexia, vomiting, diarrhea, abdominal pain, and weight loss (3). Due to non-specific clinical signs, most SEP cases are identified after exploratory laparotomy or autopsy. Particularly in cats, there is a strong tendency to conceal clinical symptoms, making it challenging to ascertain the course of the condition until severe symptoms manifest. This case presented with only abdominal distension, lethargy, and anorexia.

SEP can be diagnosed histopathologically. In humans, a staging system has been proposed for SEP; it is categorized into four stages: stage 1 (pre-SEP), stage 2 (inflammatory), stage 3 (encapsulated), and stage 4 (chronic). Staging is based on the degree of abdominal symptoms, inflammation, and encapsulation. In stage 1, the abdominal symptoms and inflammation are mild, and no encapsulation is observed. Stage 2 is characterized by the presence of abdominal symptoms including nausea, diarrhea, and mild-to-severe inflammation. Encapsulation initiates partially. In stage 3, periodic ileus is evident and inflammation is mild, but the encapsulation becomes clear. Stage 4 is characterized by persistent ileus, and chronic encapsulation. Different therapeutic and management approaches have been proposed according to the disease stage. In the early stages (stages 1–2), when inflammation is more prominent but sclerosis is minimal, corticosteroids are recommended after ruling out infection. In later stages (stages 3–4), an advanced sclerosing pattern may be present and patients may show signs of partial or complete bowel obstruction. In such cases, opioids can be used to manage abdominal pain, and tamoxifen may be administered as an anti-fibrotic drug. If symptoms are severe and the patient does not respond to medical therapy, surgical intervention may be considered (2). In the present case, persistent ileus secondary to chronic inflammation was detected. Advanced encapsulation was confirmed through CT and exploratory laparotomy. According to human staging, the patient was diagnosed with stage 4 disease, and tamoxifen administration and surgical intervention were performed.

SEP can be classified as primary or secondary, with secondary cases most often caused by diseases that trigger systemic inflammation. The reported causes of SEP in cats include a history of surgery, bacterial peritonitis, pancreatitis, foreign body ingestion, intestinal rupture, and tumors (1, 5, 12). While three types of tumors–scrotal leydigoma, duodenal leiomyosarcoma, and pancreatic adenocarcinoma– have been reported in dogs, none have previously been reported in cats (8, 12). In the present report, a cat with recurrent abdominal effusion was diagnosed with SEP via exploratory laparotomy. However, even after surgery, the clinical symptoms worsened despite treatment aimed at preventing the progression of peritonitis, and the patient died 31 days after the hospital visit. Subsequently, necropsy, histopathology, and IHC confirmed the pancreatic tumor of presumed ductal origin. Furthermore, the autopsy revealed tumor metastases to organs, including the liver, peritoneum, and lungs. More than 70% of the abdominal fat in this patient exhibited steatitis, which is believed to be the result of the systemic spread of lipolytic enzymes caused by the pancreatic tumor. Extensive steatitis of the abdominal fat and peritonitis restricted access to the pancreas, delaying definitive diagnosis.

Initially, pansteatitis was suspected based on FNA and abdominal ultrasonographic findings. Feline pansteatitis is a nutritional disease characterized by marked inflammation of fat tissue and the deposition of ceroid pigments in adipocytes resulting from vitamin E deficiency. Diseases known as ‘pansteatitis’ or ‘yellow fat disease’ improve with vitamin E administration, as vitamin E protects cells against lipid peroxidation. Moreover, in feline pansteatitis, anti-inflammatory corticosteroids are used to control pain and fever arising from inflammation of the subcutis (11). However, despite excluding infection and prescribing vitamin E along with anti-inflammatory doses of PDS, the ongoing presence of significant ascites and the lack of improvement in systemic inflammation led to the inference that there might be another underlying condition causing the inflammation.

The present case was subsequently diagnosed with presumed PDAC based on histopathological examination. PDAC has a high metastasis rate of 32–50% and a poor prognosis in cats. In a previous study, the overall prognosis was guarded, with a median survival time of 97.5 days (10). However, patients with abdominal effusion at the time of diagnosis survive for a median of 30 days (10). Moreover, pancreatic tumors exhibit many overlapping clinical symptoms with pancreatitis, such as loss of appetite, vomiting, and abdominal pain. Therefore, pancreatic tumors are often misdiagnosed as pancreatitis, and pancreatitis itself may mask concurrent tumors, causing delayed diagnosis. The present case was also presumed to have pancreatitis based on abdominal ultrasonographic findings and the feline pancreatic lipase value of the peritoneal fluid. Additionally, panniculitis or steatitis may accompany these conditions due to the systemic spread of lipolytic enzymes (10, 11).

Histopathology revealed extensive pancreatic steatosis (fatty infiltration of the pancreatic tissue) in the cat. In veterinary medicine, the relationship between pancreatic steatosis and pancreatic tumors is unclear. Recent human studies have shown that the more severe the pancreatic fat infiltration, the higher the possibility of pancreatic cancer (9). The degree of fatty infiltration in the non-cancerous parts of patients with PDAC and non-PDAC who had undergone pancreatoduodenectomy demonstrated that the degree of pancreatic fatty infiltration in patients with PDAC is significantly higher than that in non-PDAC controls (9). Therefore, an association between the severe fat infiltration in the pancreas and the pancreatic tumors in this cat can be assumed. However, further studies are required to elucidate this association.

The relationship between SEP and pancreatic tumors remains unknown. Pancreatic fibrosis, similar to that previously reported in cats with pancreatic adenocarcinoma was observed in this cat (10). According to human reports, PDAC exhibits clear pancreatic fibrosis, which can lead to SEP (13). Additionally, a dog with SEP caused by PDAC showed distinct pancreatic fibrosis (8). Therefore, it can be inferred that SEP also occurs in cats due to pancreatic fibrosis caused by PDAC, as reported in humans and dogs. Furthermore, according to reports of feline pancreatic tumors, 16/34 patients exhibited abdominal effusion (10). However, these reports did not provide detailed descriptions of the massive abdominal effusions. Therefore, it is challenging to determine whether the recurrent and massive effusions are due to the tumor or an inflammatory response. In this case, given the characteristics of the effusion, the large amount of effusion before death is presumed to have occurred due to multiple reasons. In veterinary medicine, cases of SEP have shown a variety of characteristics of peritoneal effusion depending on the underlying causes (1, 5, 6, 8). The cat in this report exhibited clear signs of inflammation, such as pansteatitis and peritonitis, based on hematological findings and exploratory laparotomy. However, the peritoneal effusion continued to present as a modified transudate. Most modified transudates result from some type of obstruction to venous or lymphatic drainage (14). In the second CT scan of this patient, a filling-defect was identified in the splenic vein, which we presumed to be due to compression from a mass-like lesion nearby. Therefore, in this patient, it is presumed that the modified transudate occurred due to congestion in the splenic vein. However, in this case, the relationship between the tumor and SEP was not clear, and both conditions can cause ascites. In the initial stage of SEP, an inflammatory response of the peritoneum leads to the formation of a ‘neo-membrane’ rich in fibrin and vessels, which induces exudate from hyperpermeable peritoneal vessels. As SEP progresses to its late stage, the thickening of the peritoneum due to inflammation and fibrosis causes compression of peritoneal vessels, reducing blood supply to the peritoneum. This impairs the proper reabsorption of fluid from the peritoneum, leading to its accumulation in the abdominal cavity and the development of ascites (15). Tumors are known to cause effusion through various mechanisms, including the secretion of vascular endothelial growth factor and other pro-angiogenic cytokines, compression of adjacent vascular and lymphatic structures, and direct invasion of blood vessels or lymphatics (14, 16). Therefore, we concluded that the interaction between these two factors might have accelerated the formation of the effusion. From an epidemiological standpoint, SEP is a chronic disease with few clinical symptoms, whereas PDAC is a highly aggressive tumor. Inflammation is a critical factor in the development and progression of pancreatic cancer (17). In this patient, because ascites of unknown origin suddenly emerged and the patient died unexpectedly 31 days after presentation, SEP may have appeared initially, whereas the patient died because of the presumed PDAC.

The diagnosis of SEP is often delayed, frequently leading to a poor prognosis. In this case, the delayed diagnosis of SEP led to a poor prognosis for the patient for the following reasons. First, the lack of awareness regarding the association between presumed PDAC and SEP meant that it took time to consider the possibility of SEP due to the tumor. The process of excluding all other conditions during the diagnosis of SEP is also time consuming. Second, the patient was initially diagnosed with feline pansteatitis based on findings and symptoms associated with this condition in FNA tests. Consequently, the patient was treated for known feline pansteatitis. However, the poor response to treatment created the necessity of considering other diagnoses. A subsequent postmortem examination revealed feline presumed PDAC as the primary cause, suggesting the pansteatitis might have been secondary to the tumor. Considering the rapid deterioration of clinical symptoms, it is plausible that the occurrence of clinical symptoms was related to the tumor. Third, as mentioned earlier, the rapid deterioration of clinical symptoms and the initial onset of systemic symptoms led to lower suspicion of a tumor. However, considering the short median survival time and high malignancy of feline PDAC as well as the presence of ascites, it is reasonable to consider PDAC as the cause. Furthermore, in this case, as no surgical biopsy was taken at laparotomy, the diagnosis of pancreatic cancer was delayed. Intense diagnostic procedure and treatments for pancreatic cancer might be required for a more favorable outcome, although this could not be determined in the present case.

Tamoxifen, a selective estrogen receptor modulator, was administered because of its supportive effect on metalloproteinase synthesis, promotion of mesothelial healing, and assistance in preventing the formation of new fibrous adhesions in the visceral peritoneum (18). Because there was a report of a dog with a traumatic penetrating abdominal injury being successfully treated for SEP with methylprednisolone and tamoxifen, tamoxifen was administered with reference to the drug dose administered in dogs (19). However, the patient died 3 days after drug administration, making it difficult to evaluate the treatment response and side effects.

SEP and PDAC are rare and only partially understood in cats. However, based on the clinical characteristics and histopathological examinations of SEP and PDAC, it is reasonable to infer a relationship between these two diseases. In conclusion, this case report describes a cat with SEP and presumed PDAC, along with concurrent massive metastasis to other organs. Further studies are necessary to ascertain the association between PDAC and SEP.

## Data availability statement

The original contributions presented in the study are included in the article/Supplementary material, further inquiries can be directed to the corresponding author.

## Ethics statement

Ethical review and approval were not required for this animal study because the case report was a retrospective evaluation with no active interventional or research components. Written informed consent was obtained from the owners for the participation of their animals in this study.

## Author contributions

CK: Conceptualization, Formal analysis, Investigation, Methodology, Visualization, Writing – original draft. SaK: Formal analysis, Methodology, Visualization, Writing – original draft. JP: Investigation, Methodology, Resources, Writing – review & editing. DL: Investigation, Methodology, Resources, Writing – review & editing. YC: Investigation, Methodology, Writing – review & editing. TY: Conceptualization, Writing – review & editing. DC: Conceptualization, Writing – review & editing. B-TK: Conceptualization, Writing – review & editing. SL: Writing – review & editing. SoK: Funding acquisition, Investigation, Methodology, Visualization, Writing – review & editing. HK: Conceptualization, Funding acquisition, Methodology, Supervision, Writing – review & editing.
