# Supplementary material for: Case report: Sclerosing encapsulating peritonitis in a cat with disseminated pancreatic adenocarcinoma of presumed ductal origin
Source: Front Vet Sci. 2024 Jul 31;11:1406223. doi: 10.3389/fvets.2024.1406223 (PMC11322092; doi:10.3389/fvets.2024.1406223)
Supplement: Supplementary file 1 [file Data_Sheet_1.PDF]

# *Supplementary Material*

Supplementary Figures

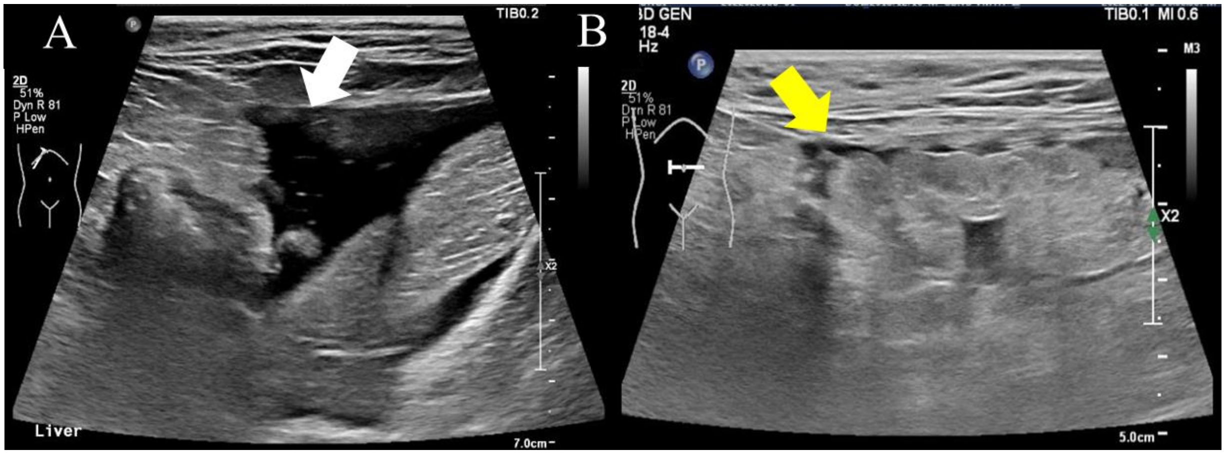


**Supplementary Figure 1.** Findings of the first abdominal ultrasound of a 9-year-old cat presenting with recurrent ascites of unknown etiology. A large amount of ascites was observed between the liver lobes, around the kidneys, and in the anterior bladder (white arrow) (A). The mesentery and fat were hyperechoic and edematous (yellow arrow) (B). The left leg of the pancreas was not clearly visible, and the pancreatico-duodenal lymph node was enlarged.

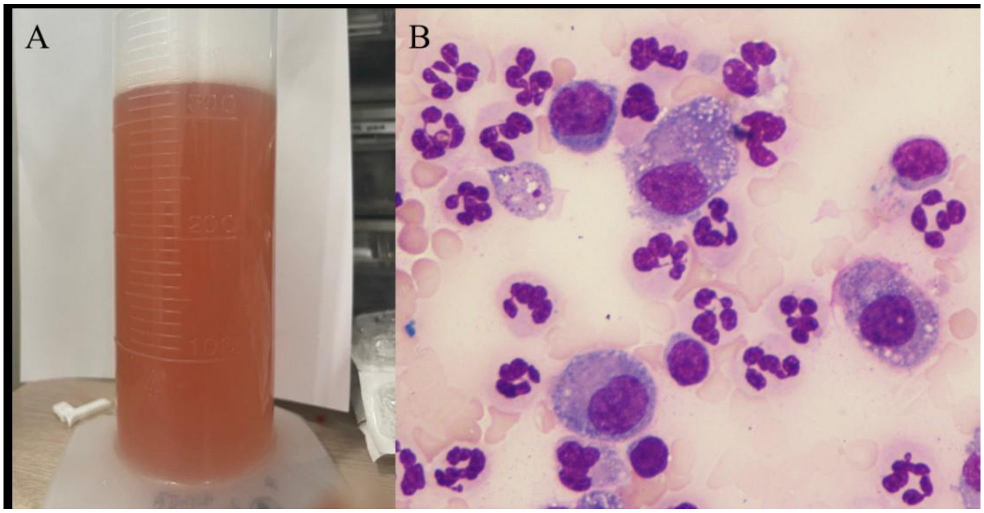


**Supplementary Figure 2**

Findings of the first peritoneal fluid sample in a cat after being referred. Approximately 320 mL of peritoneal fluid was drained via ultrasound-guided abdominocentesis. The peritoneal fluid appeared reddish and cloudy upon macroscopic examination (A). Photomicrograph of direct smear preparation of the peritoneal fluid. A variety of inflammatory cells were observed, with macrophages and

neutrophils being predominant and some lymphocytes also observed (Wright-Giemsa stain; ×1000) (B).

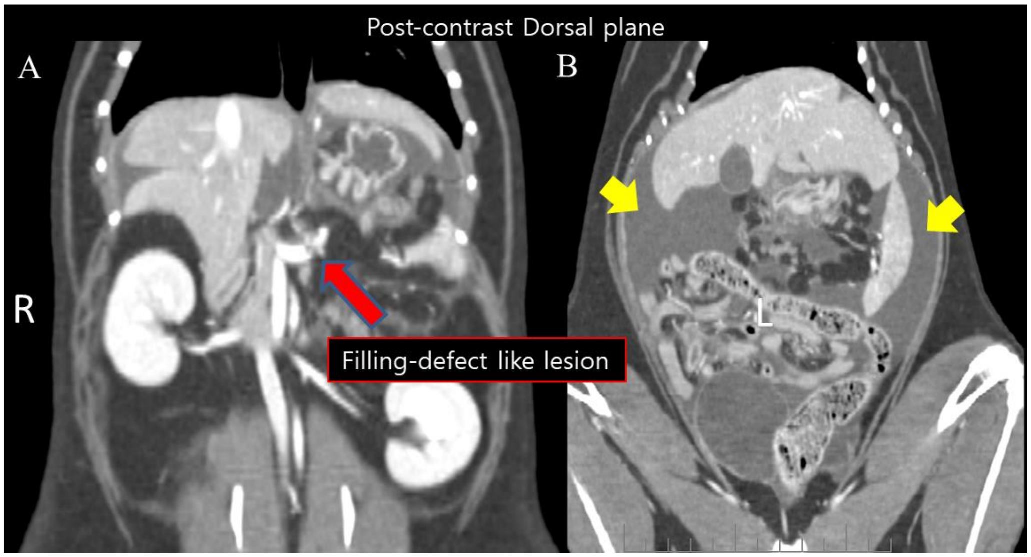


**Supplementary Figure 3.** Findings of the first computed tomography scan of a 9-year-old cat presenting with recurrent ascites of unknown etiology. Triphasic images from delay phase in the dorsal plane (A, B). A filling-defect-like lesion was observed in the blood vessels of the cranio-lateral region of the mass. Possible compression of the splenic vein by the mass was considered (red arrow). A large volume of free fluid was observed in the abdominal cavity (yellow arrow)

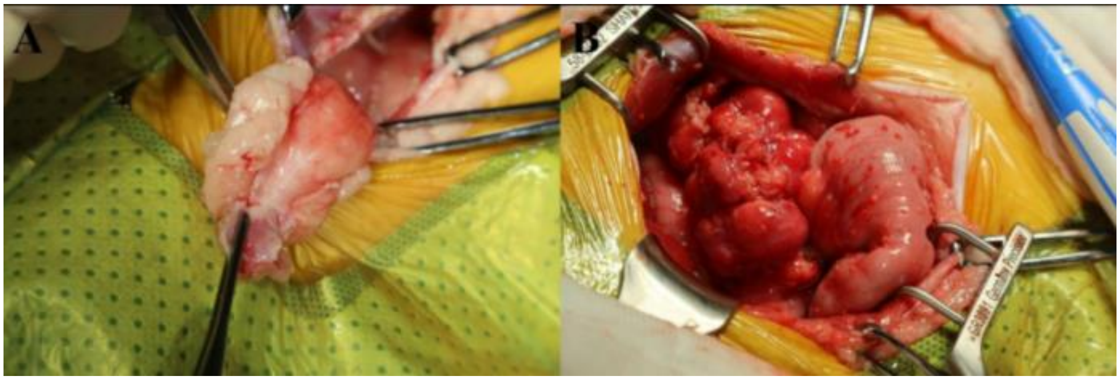


**Supplementary Figure 4.** Gross findings at laparotomy. (A) The falciform ligament was sclerosing and thickened. (B) Fibrinous adhesion of the thickened abdominal wall and organs was observed along with a large amount of ascites.

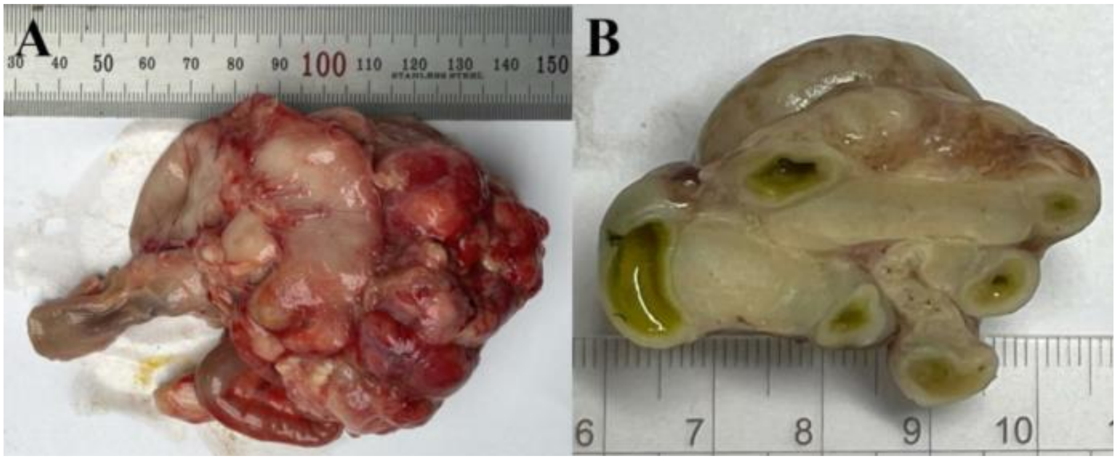


**Supplementary Figure 5.** Gross findings at necropsy. (A) Large and small intestines showed adhesion and sclerosis with numerous disseminated seeding nodules of various sizes (1–5 mm in diameter) on the surface of the serosa. (B) Tight fibrosis and steatosis between the intestines were compressing the intestines.
